# Freezing of cell sheets using a 3D freezer produces high cell viability after thawing

**DOI:** 10.1016/j.bbrep.2021.101169

**Published:** 2021-11-08

**Authors:** Koji Ueno, Soichi Ike, Naohiro Yamamoto, Yutaro Matsuno, Hiroshi Kurazumi, Ryo Suzuki, Shunsaku Katsura, Bungo Shirasawa, Kimikazu Hamano

**Affiliations:** a Department of Surgery and Clinical Science, Yamaguchi University Graduate School of Medicine, Ube, Japan; b Department of Medical Education, Yamaguchi University Graduate School of Medicine, Ube, Japan

**Keywords:** Cell sheet, Freezing, 3D freezer, Survival, Regenerative medicine, Temperature, CLI, Critical limb ischemia, ELISA, Enzyme-linked immunosorbent assay, HGF, Hepatocyte growth factor, IND, Investigational new drug, PAD, Peripheral arterial disease, TGF, Transforming growth factor, VEGF, Vascular endothelial growth factor

## Abstract

In cell therapy, transplanting an appropriate number of cells to the target site is crucial. One way to achieve this is to transplant cell sheets. Transplantation of cell sheets has already been utilized for various diseases in clinical practice. However, reducing the cost of cell sheet utilization is essential so as to facilitate the spread of regenerative medicine. Several ways to reduce costs are available, one of which is the use of allogenic cells. Another alternative is the use of cell sheets, which necessitates the development of methods for freezing cell sheets. This is the first study to report the use of a 3D Freezer for freezing cells. 3D Freezers have been used in the field of food processing and technology for a long time. The 3D Freezer freezes objects using cold air at a uniform temperature from all directions. In this study, we analyzed the cooling speed of human fibroblast sheets in 11 cell preservation solutions using a 3D Freezer and a Program Freezer. The cooling speed was −2 °C per min in the 3D Freezer. Supercooling in 10 cell preservation solutions was lower in the 3D Freezer than in the Program Freezer. Cell viability after freeze–thaw of the cell sheets using 3D Freezer was more than 70% in five cell preservation solutions. The levels of hepatocyte growth factor and transforming growth factor-β1 were the same not only in the fibroblast sheets frozen using the five cell preservation solutions but also in the non-frozen fibroblast sheets. These results suggest that the 3D Freezer can freeze implantable cell sheets immediately after thawing.

## Highlights

• 3D Freezer operates using cold air with uniform temperature directed at an object from all directions.
• 3D Freezer was trialed for freezing 3D cell sheets for the first time.
• 3D Freezer can generate high cell viability after freezing and thawing for cell therapy.

## 1 Introduction

Peripheral arterial disease (PAD) is an ischemic disease caused by arteriosclerosis. Approximately 1%–3% of patients with PAD are diagnosed with critical limb ischemia (CLI). Of these patients, 40% have a limb amputated within a year despite undergoing treatment because the ischemia did not improve [1]. Clinical trials of cell therapy have been conducted to promote angiogenesis in CLI. Symptoms, including ulcers, showed improvement in some cases [2]. Single cell suspensions have been applied to the legs of patients with CLI using intramuscular injections. In addition, transplantation of cell sheets has been reported to facilitate efficient engraftment at the transplant site [3]. Cells transplanted in the form of cell sheets were believed to result in more effective treatment of ulcers compared with the transplantation of single cell suspensions.

We reported that transplantation of autologous mixed-cell sheets may be a novel therapeutic method using an animal ulcer model [[4], [5], [6]]. Autologous mixed-cell sheets were prepared by co-culturing two cell types using temperature-responsive culture dishes (UpCell, CellSeed, Tokyo, Japan). One cell type was peripheral blood mononuclear cells, which were responsible for the secretion of growth factors and cytokines. The other cell type was fibroblasts, responsible for the secretion of the extracellular matrix, which became a substrate for the cell sheets. To improve handling of the cell sheets, we developed a simple method to manufacture multilayered cell sheets and reported the usefulness of autologous multilayered mixed sheets composed of peripheral blood mononuclear cells and fibroblasts in a mouse ulcer model [7]. To reduce manufacturing costs and eliminate the time lag until transplantation for clinical application of the cell sheets, allogeneic multilayered fibroblast sheets were used in the mouse ulcer model. Allogeneic multilayered fibroblast sheets showed the same therapeutic effect as autologous multilayered fibroblast sheets [8].

Cryopreservation of cell sheets can help reducing the manufacturing costs and make cell sheet transplantation more convenient. However, cells are usually frozen as single cell suspensions using a device such as a Program Freezer [9]. 3D Freezers (Koga Sangyo Co., Ltd., Shimonoseki City, Japan) are used for freezing in the field of food processing and technology. For the purpose of freezing, this freezer uses cold air with uniform temperature directed at an object from all directions. Therefore, a 3D Freezer was used to investigate the methods available for freezing cell sheets, which are three-dimensional structures, in this study.

## 2 Materials and methods

### 2.1 Cells

Human fibroblasts were isolated from the oral tissues of healthy volunteers and cultured with equal amounts of CTS™ AIM V ™ SFM medium (Thermo Fisher Scientific, Waltham, MA, USA) and HFDM-1 (+) (Cell Science & Technology Institute, Sendai, Miyagi, Japan) supplemented with 2% NeoSERA (Japan Biomedical Co., Ltd., Otofune, Hokkaido, Japan) at 37 °C in a humidified 5% CO_2_ incubator. This study was approved by the Institutional Review Board Committee of Yamaguchi University Hospital (#H26-111) and was conducted in accordance with the Declaration of Helsinki. Informed consent was obtained from all study participants prior to their participation in the study.

### 2.2 Measurement of cooling temperature of cell preservation solutions

A 2-mL aliquot of cell preservation solution per well was transferred into each of three wells in a six-well plate. The tips of a temperature sensor were immersed in the cell preservation solution. The plates were placed in a 3D Freezer (Koga Sangyo Co., Ltd.) or a Program Freezer (STREX Inc., Osaka Japan). The temperature was analyzed using a temperature measuring device (#ADL12, AS ONE CORPORATION, Osaka, Japan). The cell preservation solutions used were as follows: STEM-CELLBANKER® GMP grade and STEM-CELLBANKER® DMSO Free GMP grade (ZENOAQ RESOURCE CO., LTD., Koriyama, Fukushima, Japan); Bambanker® hRM, Bambanker, and Bambanker DMSO Free (Nippon Genetics Co., Ltd., Tokyo, Japan); Cell Reservoir One (without DMSO) (Nacalai Tesque, Inc., Kyoto, Japan); Cryo Scarless DMSO Free, Stem Cell Keep, and ThelioKeep (BioVerde, Kyoto, Japan); Cellvation (Protide Pharmaceuticals, Inc., Lake Zurich, IL, USA); and Repro Cryo RM (REPROCELL, Yokohama, Kanagawa, Japan).

### 2.3 Fibroblast sheets

Fibroblasts were seeded in a 24-well plate (5.0 × 10^5^ cells/2 mL medium/well) for cell viability (three wells for each cell preservation solution) and a 12-well plate (1.0 × 10^6^ cells/4 mL medium/well) to investigate freeze–thaw using equal amounts of the medium containing CTS™ AIM V™ SFM and HFDM-1 (+) supplemented with 2% NeoSERA at 37 °C in a humidified 5% CO_2_ incubator for 3 days. Fibroblasts were incubated with 10 PU/mL dispase (GODO SHUSEI CO., LTD., Tokyo, Japan) and peeled using tweezers to prepare fibroblast sheets.

### 2.4 Freezing and thawing method

Fibroblast sheets were soaked in 300 μL of cell preservation solution per well in 24-well plates and 1000 μL of cell preservation solutions per well in 12-well plates. For freezing using the 3D Freezer, multi-well plates were placed in a precooled 3D Freezer at −35 °C for 20 min. Multi-well plates were stored in a −80 °C freezer for 2 h. For freezing using the Program Freezer, the parameters for cooling were as follows: 4 °C for a 5-min hold, −2 °C per min down to −30 °C, −30 °C for a 5-min hold, and −1 °C per min down to −80 °C. Multi-well plates were stored in a −80 °C freezer for 2 h. For thawing the cells, the multi-well plates were placed on a Thermo plate (Tokai Hit., Co., Ltd., Shizuoka-ken, Japan) at 37 °C for 14 min. Fibroblast sheets were soaked in phosphate-buffered saline (PBS) (Cell Science & Technology Institute) twice for washing. To measure cell viability, fibroblast sheets were incubated in a 24-well plate (1 mL medium/well) with equal amounts of CTS™ AIM V™ SFM and HFDM-1 (+) medium supplemented with 2% NeoSERA at 37 °C in a humidified 5% CO_2_ incubator for 3 days. The supernatant was collected for enzyme-linked immunosorbent assay (ELISA). Non-frozen fibroblast sheets were soaked in PBS and stored at room temperature instead of freezing and storing at −80 °C.

### 2.5 Cell viability

Fibroblast sheets were soaked in 500 μL of a 1:4 mixture of (3-(4,5-dimethylthiazol-2-yl)-5-(3-carboxymethoxyphenyl)-2-(4-sulfophenyl)-2H-tetrazolium) (MTS) reagent (CellTiter 96 Aqueous One Solution Cell Proliferation Assay; Promega, Madison, WI, USA) and medium at 37 °C for 4 h. After incubation, 100 μL of the supernatant from each well was transferred to a 96-well plate and its absorbance was measured at 490 nm on a microplate reader (2030 ARVO X4; PerkinElmer, Boston, MA, USA).

### 2.6 Enzyme-linked immunosorbent assay

ELISA was performed using R&D Systems® ELISA Kits (# DHG00B and # DB100B, R&D Systems, Minneapolis, MN, USA) to measure the concentration of hepatocyte growth factor (HGF) and transforming growth factor-β1 (TGF-β1) in the supernatant after the cell sheets were frozen and thawed, followed by 3 days of incubation. Because the medium was supplemented with NeoSERA, which is a type of bovine serum, the TGF-β1 concentration of the medium was measured and this value was then subtracted from the TGF-β1 values obtained for the supernatant from the wells in which fibroblast sheets were cultured.

### 2.7 Statistical analysis

The results were presented as mean ± standard deviation. All statistical analyzes were performed using GraphPad Prism version 8 (GraphPad Software, San Diego, CA, USA; www.graphpad.com). Statistical significance was assessed using Ordinary one-way ANOVA multiple comparison with non-frozen cells as controls. A p-value of < 0.05 was considered to indicate statistical significance: *p < 0.05; **p < 0.01; ***p < 0.001 and ****p < 0.0001.

## 3 Results

### 3.1 Freeze–thaw survival of fibroblasts attached to the bottom of the cell culture dishes

**Fig. 1:**
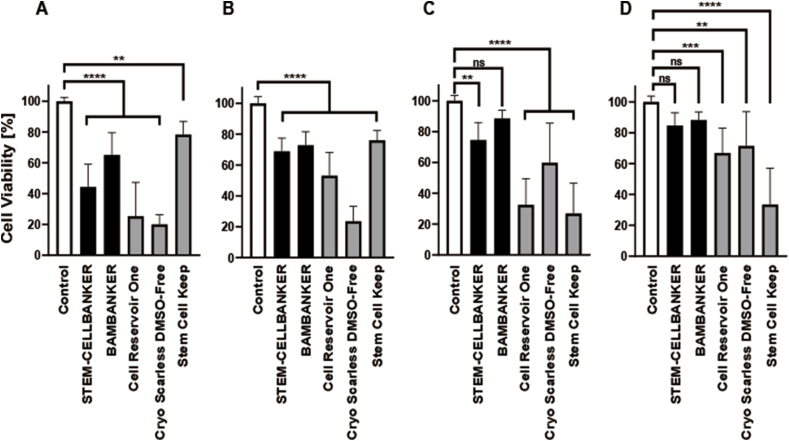
Cell viability of adherent fibroblasts after freeze–thaw. (A) Adherent fibroblasts were frozen in five cell preservation solutions using a Program Freezer and the viability of the adherent fibroblasts was evaluated using (3-(4,5-dimethylthiazol-2-yl)-5-(3-carboxymethoxyphenyl)-2-(4-sulfophenyl)-2H-tetrazolium) (MTS) 24 h after thawing (n = 9). (B) Adherent fibroblasts were frozen in five cell preservation solutions using a Program Freezer and the viability of the adherent fibroblasts was evaluated using MTS 3 days after thawing (n = 9). (C) Adherent fibroblasts were frozen in five cell preservation solutions using a 3D Freezer and the viability of the adherent fibroblasts was evaluated using MTS 24 h after thawing (n = 9). (D) Adherent fibroblasts were frozen in five cell preservation solutions using a 3D Freezer and the viability of the adherent fibroblasts was evaluated using MTS 3 days after thawing (n = 9). The white bars represent control non-frozen cells. The black bars represent cell preservation solutions including dimethyl sulfoxide (DMSO). The gray bars represent cell preservation solutions not containing DMSO.

To observe cell viability after using different freezing devices, the fibroblasts were seeded and incubated in a 24-well plate. Medium was replaced with several types of cell preservation solutions and cells were frozen using either a 3D Freezer or a Program Freezer. Cell survival experiments were conducted at 24 h and 3 days after thawing. Stem Cell Keep showed the highest survival rate for cells out of all the other cell preservation solutions in a Program Freezer (Fig. 1A and B). A significant difference in cell viability was observed when Stem Cell Keep was compared with control. Bambanker showed no significant difference in cell viability compared with control in a 3D Freezer (Fig. 1C). STEM-CELLBANKER and Bambanker showed no significant difference in cell viability compared with control in a 3D Freezer (Fig. 1D).

### 3.2 Analysis of the cooling rate of the cell preservation solution

**Fig. 2:**
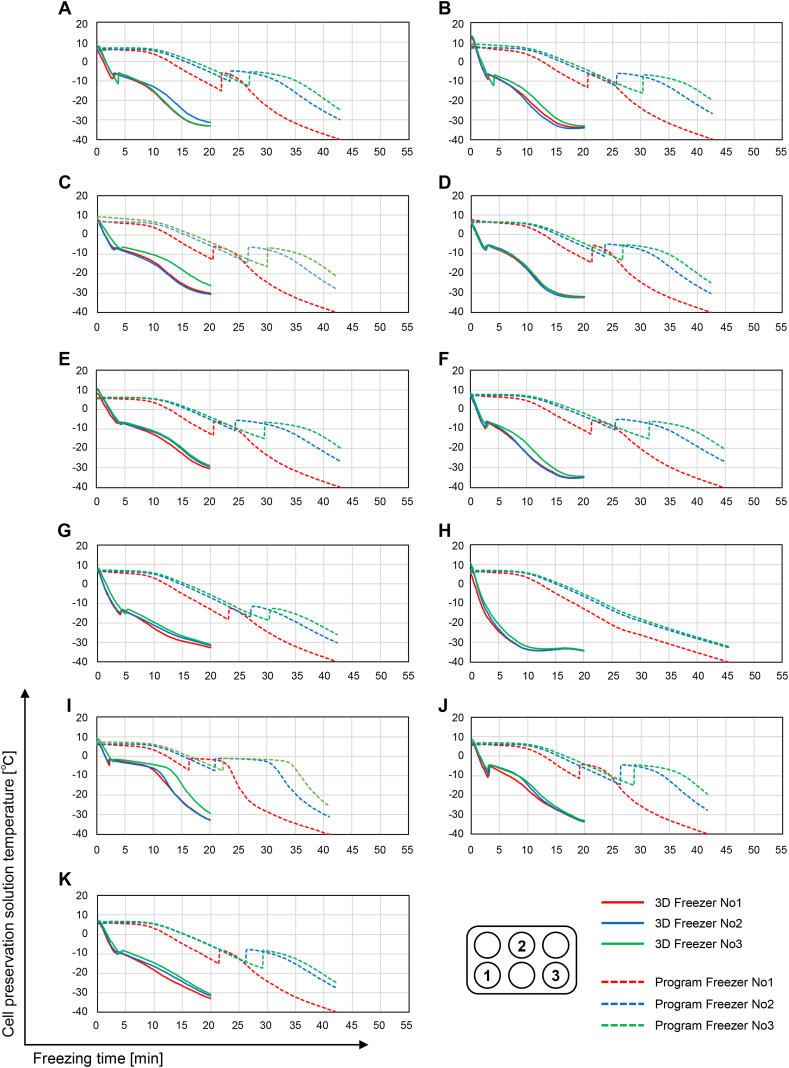
Measurement of cooling temperature in cell preservation solutions in a 3D Freezer and a Program Freezer. Eleven cell preservation solutions were applied to three wells in six-well plates marked with numerical characters and frozen using a 3D Freezer or a Program Freezer. The cooling temperature was measured. The solid line indicates cell preservation solutions frozen using the 3D Freezer. The dotted line indicates cell preservation solutions frozen using the Program Freezer. The cell preservation solutions used were as follows: (A) STEM-CELLBANKER; (B) Bambanker hRM; (C) Bambanker; (D) STEM-CELLBANKER DMSO Free; (E) Cell Reservoir One (without DMSO); (F) Cryo Scarless DMSO Free; (G) Bambanker DMSO Free; (H) Stem Cell Keep; (I) ThelioKeep; (J) Cellvation; (K) Repro Cryo RM.

To analyze the cooling rate of the cell preservation solution caused by the difference in the freezing devices, cell preservation solutions were added to six-well plates, the multi-well plates were then placed in a freezing device, and the freezing temperature was recorded. The temperature of the 11 cell preservation solutions placed in the 3D Freezer reached approximately −30 °C in 20 min because the preset temperature for this device was −35 °C. The temperature of the 11 cell preservation solutions placed in the Program Freezer reached approximately −30 °C in about 40 min because freezing is carried out slowly by this device. Its cooling rate is −3 °C to −1 °C per min. No supercooling was observed when Stem Cell Keep was used for cooling in either the 3D Freezer or the Program Freezer, but the other 10 cell preservation solutions caused supercooling. Because the cooling speed of the 3D Freezer was faster than that of the Program Freezer, supercooling in the 10 cell preservation solutions, excluding Stem Cell Keep, was lower in the 3D Freezer than in the Program Freezer (Fig. 2).

### 3.3 Freeze–thaw survival and secretion of growth factors in fibroblast sheets

**Fig. 3:**
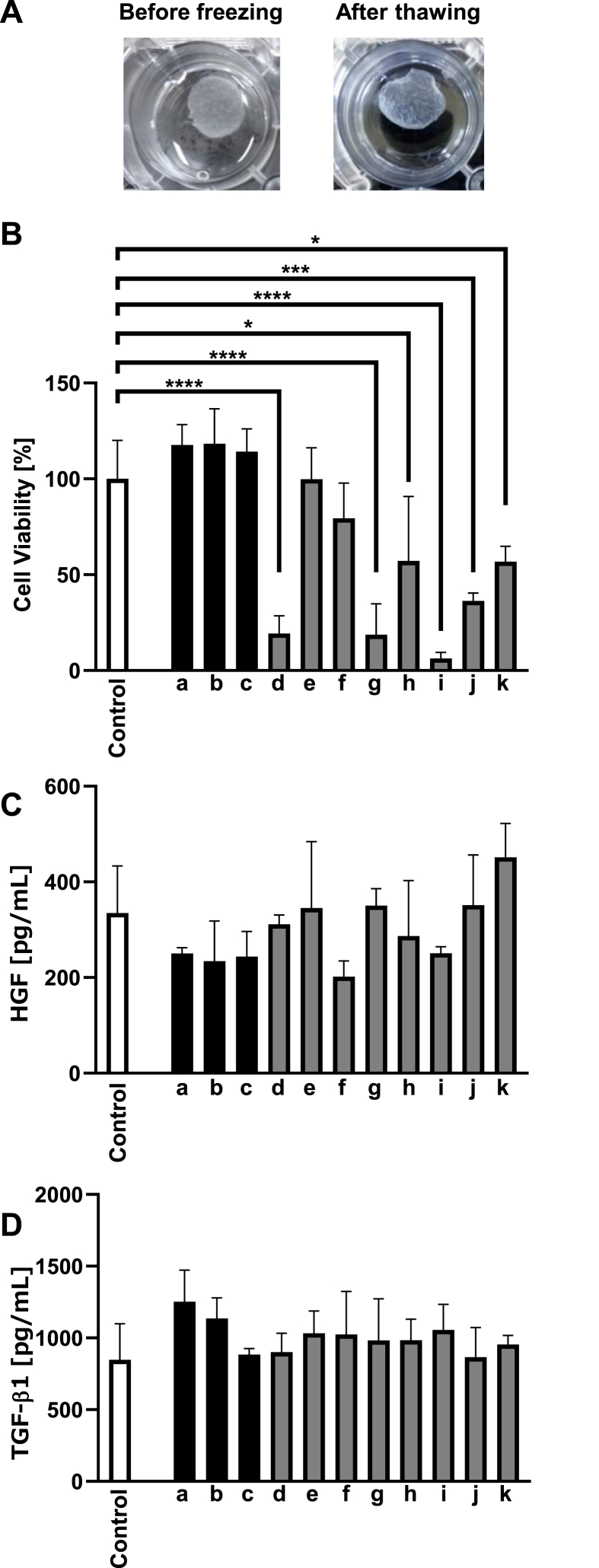
Cell viability and the ability of fibroblast sheets to secrete growth factors after freeze–thaw. (A) Morphological observation of fibroblast sheets before freezing and after thawing. A fibroblast sheet was prepared in a 12-well plate and soaked into Bambanker hRM (left side). The fibroblast sheet was frozen using a 3D Freezer, thawed and placed in phosphate-buffered saline (PBS) (right side). (B) The fibroblast sheets were frozen in 11 cell preservation solutions using a 3D Freezer and cultured for 3 days after thawing, and the cell viability of the fibroblast sheet was evaluated using (3-(4,5-dimethylthiazol-2-yl)-5-(3-carboxymethoxyphenyl)-2-(4-sulfophenyl)-2H-tetrazolium) (MTS) (n = 3). The cell preservation solutions used were as follows: (a) STEM-CELLBANKER; (b) Bambanker hRM; (c) Bambanker; (d) STEM-CELLBANKER DMSO Free; (e) Cell Reservoir One (without DMSO); (f) Cryo Scarless DMSO Free; (g) Bambanker DMSO Free; (h) Stem Cell Keep; (i) ThelioKeep; (j) Cellvation; (k) Repro Cryo RM. (C) The hepatocyte growth factor (HGF) concentration was analyzed in the supernatant of fibroblast sheets cultured for 3 days after thawing (n = 3). (D) The transforming growth factor-β1 (TGF-β1) concentration was analyzed in the supernatant of fibroblast sheets cultured for 3 days after thawing (n = 3). The white bars indicate control non-frozen cell sheets. The black bars indicate cell preservation solutions including dimethyl sulfoxide (DMSO). The gray bars indicate cell preservation solutions not containing DMSO.

To confirm cell viability of the cell sheets after freeze–thaw, fibroblasts were peeled from the bottom of the wells into multi-well plates, and fibroblast sheets were prepared (Fig. 3A). Fibroblast sheets were immersed in the cell preservation solutions and frozen in the 3D Freezer followed by storage at −80 °C. The frozen fibroblast sheets were thawed (Fig. 3A) and cultured for 3 days to investigate whether the freeze-thawed fibroblast sheets were alive and secreting growth factors. There was no significant difference in cell viability among the cell sheets frozen in STEM-CELLBANKER, Bambanker hRM, Bambanker, Cell Reservoir One (without dimethyl sulfoxide (DMSO)), or Cryo Scarless DMSO Free compared with non-frozen controls (Fig. 3B). The concentration of HGF and TGF-β1 was the same in all frozen and thawed fibroblast sheets compared with control (Fig. 3C and D).

## 4 Discussion

Cell sheet transplantation is a promising cell transplantation method for use in regenerative medicine [3]. The transplantation of fibroblast sheets was found to be useful not only in a mouse ulcer model [7] but also in the prevention of postoperative complications in a rat model [10,11]. Cardiac function also reportedly improved after the transplantation of cardiosphere-derived cell sheets in mouse and rabbit models [12,13] and mesenchymal stem cell sheets in a rabbit model [14]. Cell sheet transplantation is used at present in both clinical trials and in clinical practice [[15], [16], [17], [18]]. Although conventional cell sheet transplantation uses autologous cell sheets, reducing manufacturing costs is essential so as to improve the feasibility of treatment using cell sheets. One way of reducing costs is transplantation of allogenic cell sheets rather than autologous cell sheets used in tailor-made medicine. In the current cell sheet manufacturing process, a cell suspension is seeded and incubated, and the resulting cell sheets are transplanted to target sites. Cryopreservation is not included in the process that extends from cell seeding to transplant because no method is available at present for freezing cell sheets for use in clinical practice. Therefore, in this study, a 3D Freezer was trialed for freezing 3D cell sheets for the first time.

Two possible methods to incorporate the cryopreservation of cell sheets in the process extending from cell seeding to transplant are available. The first method is to freeze cells before peeling them for preparing cell sheets, as shown in Fig. 1. The second is to freeze cell sheets in a state in which they can be transplanted, as shown in Fig. 3. It is suggested that the assessment of cell viability should be performed 24 h after thawing because injury to cells due to freezing and thawing induces apoptosis [9]. There was no significant difference between the cell viability of the control cells and that of the fibroblasts adhering to their respective substrates frozen in Bambanker in a 3D Freezer 24 h after thawing (Fig. 1C). Although other cell preservation solutions showed significantly lower cell viability compared with controls in both freezing devices 24 h after thawing, the cell viability in Stem Cell Keep was approximately 76% using the Program Freezer and approximately 27% using the 3D Freezer. Stem Cell Keep containing no dimethyl sulfoxide is a cell preservation solution for vitrification, not slow freezing. This result suggests that it is important to explore combinations of cell preservation solutions and freezing devices to establish their suitability for each cell type.

According to Food and Drug Administration guidelines, cell viability is required to be more than 70% for somatic cell therapies [19]. The cryopreservatives that met this criterion in frozen and thawed fibroblast sheets were STEM-CELLBANKER (117%), Bambanker hRM (118%), Bambanker (114%), Cell Reservoir One (without DMSO) (99%), and Cryo Scarless DMSO Free (79%) (Fig. 3B). These five cell preservation solutions maintained the capacity of fibroblast sheets to secrete HGF and TGF-β1 compared with the control (Fig. 3C and D). HGF and TGF-β1 are essential for angiogenesis and immune tolerance [[20], [21], [22]]. Therefore, freezable fibroblast sheets using allogenic cells, such as mesenchymal stem cells, may be useful. In this study, the preset temperature was set to −35 °C in the 3D Freezer, and multi-well plates were frozen by cold air at −35 °C with uniform temperature from all directions. Thus, the cooling speed in the cell preservation solutions was about twice as fast in the 3D Freezer than in the Program Freezer (Fig. 2). Cooling from about 10 °C to −30 °C in the 3D Freezer required 20 min. This cooling speed was approximately −2 °C per min. Usually, cooling takes more than 90 min using the Program Freezer, before the cells are transferred to a −80 °C freezer for storage. Before performing the next freezing operation, it takes more than 30 min to return the Program Freezer to 4 °C. However, cell sheets were frozen using the 3D Freezer in 20 min, followed by transfer to a −80 °C freezer for storage. The next freezing operation could be performed after a short time. The 3D Freezer can therefore contribute to high productivity of frozen cells and could generate high cell viability after freezing and thawing for cell therapy. We evaluated the viability of cell sheets using an MTS tetrazolium compound. NADPH or NADH converts the MTS tetrazolium compound into a colored formazan product and the absorbance of this colored formazan product is measured to determine the cell viability. The concentrations of HGF and TGF-β1 were not correlated with cell viability, as shown in Fig. 3. The remaining cell preservation solutions may be preventing the chemical changes such as the change from MTS tetrazolium to a colored formazan product. Therefore, our data suggest that choosing cell preservation solutions that induce both high cell viability and the secretion of growth factors/cytokines is essential when considering potential clinical applications.

The 3D Freezer makes it possible to retain high cell viability after freeze–thaw and to transplant the cell sheets immediately after thawing. Because of that, the 3D Freezer contributes toward lowering manufacturing costs of mass production especially in allogenic cell sheets. This study suggests that the 3D Freezer is a useful device for freezing three-dimensional structures such cell sheets.

## Declaration of competing interest

There are no conflicts of interest to declare.

## Data Availability

The data that has been used is confidential.
